# Adiponectin: Structure, Physiological Functions, Role in Diseases, and Effects of Nutrition

**DOI:** 10.3390/nu13041180

**Published:** 2021-04-02

**Authors:** Kayvan Khoramipour, Karim Chamari, Amirhosein Ahmadi Hekmatikar, Amirhosein Ziyaiyan, Shima Taherkhani, Nihal M. Elguindy, Nicola Luigi Bragazzi

**Affiliations:** 1 Department of Physiology and Pharmacology, Afzalipour Medical Faculty, Physiology Research Center and Neuroscience Research Center, Kerman University of Medical Sciences, Kerman 7616913555, Iran; K.khoramipour@kmu.ac.ir; 2 ASPETAR, Qatar Orthopaedic and Sports Medicine Hospital, Doha 29222, Qatar; karim.chamari@aspetar.com; 3 Physical Education & Sport Sciences Department, Faculty of Humanities, Tarbiat Modares University, Tehran 1411713116, Iran; a.ahmadihekmatik@modares.ac.ir; 4 Department of Exercise Physiology, Faculty of Physical Education and Sports Science, University of Tehran, Tehran 1439956141, Iran; A.ziyaiyan74@ut.ac.ir; 5 Department of Exercise Physiology, Faculty of Sport Sciences, University of Guilan, Rasht 4199843653, Iran; Shimataherkhani6@gmail.com; 6 Biochemistry Department, Faculty of Science, Alexandria University, Alexandria 21568, Egypt; faynt2001us@yahoo.com; 7 Laboratory for Industrial and Applied Mathematics (LIAM), Department of Mathematics and Statistics, York University, Toronto, ON M3J 1P3, Canada

**Keywords:** adiponectin, adipose tissues, adipokine, disease, biomarker, cancer, nutrition

## Abstract

Adiponectin (a protein consisting of 244 amino acids and characterized by a molecular weight of 28 kDa) is a cytokine that is secreted from adipose tissues (adipokine). Available evidence suggests that adiponectin is involved in a variety of physiological functions, molecular and cellular events, including lipid metabolism, energy regulation, immune response and inflammation, and insulin sensitivity. It has a protective effect on neurons and neural stem cells. Adiponectin levels have been reported to be negatively correlated with cancer, cardiovascular disease, and diabetes, and shown to be affected (i.e., significantly increased) by proper healthy nutrition. The present review comprehensively overviews the role of adiponectin in a range of diseases, showing that it can be used as a biomarker for diagnosing these disorders as well as a target for monitoring the effectiveness of preventive and treatment interventions.

## 1. Introduction

Adiponectin is a circulating hormone secreted by adipose tissue which exerts protective effects against inflammation and can positively modulate the endocrine system, by enhancing insulin sensitivity in obese animals as well as in humans [1,2]. Interestingly, gender seems to influence adiponectin levels: several studies have shown higher levels of adiponectin in women with respect to men probably due to higher levels of estrogen hormone, which is known to have an impact on the adipose tissue [3]. Adiponectin also plays protective roles against diabetes and atherosclerosis [4,5]. In addition, this cytokine enhances fatty acids catabolism and actively regulates blood sugar [6,7]. Adiponectin stimulates fatty acids oxidation in the skeletal muscle, and subsequently reduces triglycerides (TG) accumulation [8,9]. Its concentration has been found to be reduced in obese subjects, unlike other adipokines, probably due to low physical activity and sedentary/unhealthy lifestyles. Practicing physical activity can revert such a condition, in that it stimulates the production and release of adiponectin, which enhances glucose uptake and fatty acids oxidation through 5’-adenosine monophosphate (AMP) kinase (AMPK) activation [10,11].

From a molecular standpoint, adiponectin can be found in three forms with different molecular weights, namely, a low molecular weight (LMW), a moderate molecular weight (MMW), and a high molecular weight (HMW) form. Each form has specific activities which are described in the present article [9,12]. For example, HMW correlates with glucose uptake and central obesity [13,14,15]. HMW levels also increase after and correlate with decreasing body weight [16]. In this review, we look at adiponectin roles in different diseases, such as obesity, diabetes, cardiovascular disorders, and various forms of cancer, as well as describing its molecular and cellular cascades in different organs and its responses to diet.

## 2. Structure

**Figure 1:**
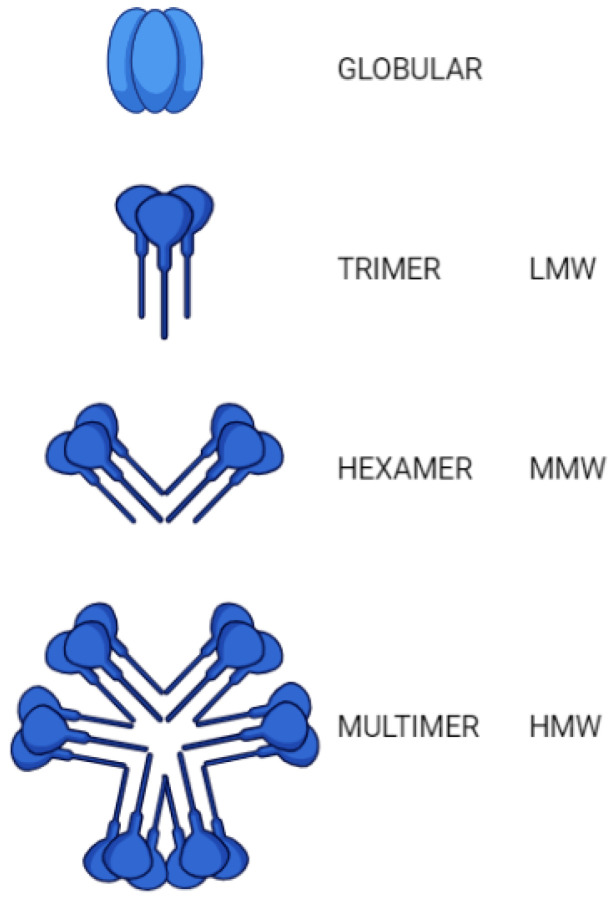
Three structural types of adiponectin with different molecular weight (trimer, hexamer, and multimer). A full-length adiponectin (~30 kDa) consists of a globular domain, a collagenous domain, a species-specific domain, and a signal peptide. Oligomerization facilitates the formation of the trimer, hexamer, and high-molecular weight (HMW) adiponectin. Full-length adiponectin can undergo proteolytic cleavage, whose proteolytic fragment corresponds to the globular adiponectin. AdipoR1 has a greater affinity for the globular form, whereas AdipoR2 has a moderate affinity for both globular and full-length forms.

Adiponectin is a protein with 224 amino acids produced by white adipose tissues (WAT) [17]. This hormone was first identified in 1995 and the gene coding for this protein is located on the 3q27 chromosome [18,19]. The adiponectin structure is made up of single-chain trimers, namely, a variable N-terminal domain, a collagen domain, and a C-terminal globular domain homologous to the immune complement C1q. Three spherical domains ranging from Pro104 to Asn244, which are positioned by the End-N and C ends (two monomers adjacent to each other), are connected by means of the Pro104-Tyr109 hinge, which serves as a link between the two monomers. This single-chain trimer is covered by a bell-shaped structure, and each spherical part contains approximately 10 twisted strings. This structure resembles the membrane structure of the proteins belonging to the C1q family and the three-dimensional structure of the proteins of the tumor necrosis factor (TNF) family. Belonging to the C1q-TNF superfamily, it has been highlighted that there is a notable similarity between the structure of the spherical part of the adiponectin and TNF-alpha (TNF-α), but their amino acid sequences are different. Adiponectin has the form of a trimer (about 90 kDa; base unit), hexamers (approximately 180 kDa, a type of LMW form), or multimer (HMW species, weighing more than 400 kDa) (Figure 1). It has been pointed out that the longitudinal shape is not generally found in normal conditions due to high thermodynamic instability, but the breakdown of protein products (containing a spherical end-spin domain) is found within the body. All these factors (functional adjustment through post translational modifications and monomeric form instability) suggest that adiponectin probably has a role in a range of human disorders due to its several levels of intrinsic instability [20].

## 3. Adiponectin Receptors

**Figure 2:**
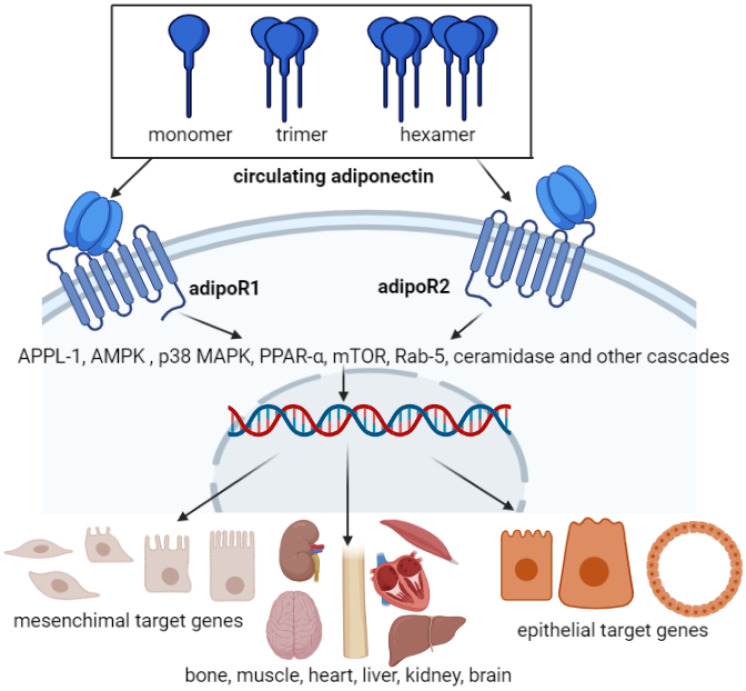
Adiponectin and its receptors and schematic representation of adiponectin-induced signaling pathway. Adiponectin interacts with the C-terminus (carboxyl end) of receptors, which interacts with the protein-adaptive protein (APPL1) in most of the seven regions with its intramuscular N terminus. T-cadherin is a receptor with a high affinity for high molecular weight adiponectin (HMW) isoforms, leading to a complex cascade of events.

Adiponectin has two major receptors, AdipoR1 and AdipoR2. Both are surface membrane proteins with seven transmembrane domains (Figure 2), with similar molecular structures, and are expressed in liver, muscle, and adipose tissue in humans. AdipoR1 is a high-affinity receptor for globular adiponectin, as well as low-affinity receptor for full-length adiponectin in skeletal muscle. In contrast, AdipoR2 is an intermediate-affinity receptor for both globular and full-length high molecular weight adiponectin forms in liver [21]. Adiponectin binds to receptors (AdipoR1 and AdipoR2) to control whole-body energy, inflammatory responses, insulin sensitivity, and fat burning process [22,23]. The presence of AdipoR1 and AdipoR2 was confirmed by siRNA studies to be essential for the binding of adiponectin to the cell membrane surface in cultured cells with loss of their binding and action shown in AdipoR1/AdipoR2 double-knockout mice, demonstrating that AdipoRs represent essential adiponectin receptors in the body. Each receptor is encoded by its own genes. In humans and mice, AdipoR1 is located on chromosomes 1p36.13-q41 and 1 E4, respectively, whereas AdipoR2 is located on chromosomes 12p13.31 and 6 F1. AdipoR1 and AdipoR2 are membrane receptors with seven transverse membrane regions and are located in a distinct position from the usual placement of receptor proteins coupled to the G protein (GPCR). Adiponectin binds to the extracellular C terminus of the adiponectin receptor, while the receptor N terminus binds to an adaptive protein (known as “adaptor protein, phosphotyrosine interacting with PH domain and leucine zipper 1” or APPL1) (Figure 2). AdipoR1 is widely expressed in skeletal muscle, synovial fibroblasts, endothelial and atrial cells, and AdipoR2 is expressed mainly in the liver, which can inactivate “Peroxisome proliferator-activated receptors type alpha” (PPAR-α) receptors that increase insulin sensitivity. Blood insulin levels can regulate AdipoRs expression [24,25]. AdipoR1 has a greater affinity for spherical adiponectin, while AdipoR2 has a higher affinity for longitudinal and other forms of adiponectin [26].

Hug et al. (2004) isolated a third adiponectin receptor, which is expressed in vascular endothelial cells and smooth muscle, by exploiting advanced expression cloning techniques. Surprisingly, this receptor is identical to a unique cadherin molecule, T-cadherin; expression is known to be correlated with progression of atherosclerosis. T-cadherin is a special receptor because it lacks cytoplasmic and transmembrane domains [27]. The precise mechanism by which T-cadherin influences intracellular signaling is unclear, but it has been suggested that this receptor may require interaction with transmembrane proteins for some physiological actions [28].

## 4. Adiponectin Functions in Different Body Organs

Adiponectin exerts pleiotropic actions, i.e., it promotes insulin sensitivity, promotes apoptosis in carcinogenic cells, and has anti-oxidant and anti-inflammatory effects. These actions could result in different effects in different organs. Below are the main organs which can be affected by adiponectin.

### 4.1. Adiponectin Functions in the Central Nervous System

Adiponectin has insulin-sensitizing, anti-inflammatory, angiogenic, and vasodilatory properties, which may affect central nervous system (CNS) disorders. Although initially not thought to pass through the blood–brain barrier, adiponectin can enter the brain through peripheral circulation and can control important brain functions, such as energy homeostasis, hippocampal neurogenesis, and synaptic plasticity. Adiponectin also controls energy, body weight, and inactivates glial cells in the brain, and thus, it prevents inflammation [29]. Adiponectin signaling cascades affect satiety and energy homeostasis and also control neurogenesis and synaptic plasticity in the hypothalamus. In addition, adiponectin stimulates proliferation in hippocampal progenitor cells and Neuro2A cells through AdipoR1 signaling [30]. Reduced adiponectin concentrations at the level of the dentate gyrus (DG) of adult male mice lead to decreased neurogenesis, and adiponectin infusion rises neurogenesis in hippocampal region [31]. This effect is controlled by activation of p38-mitogen-activated protein kinase (MAPK) and the resultant inactivation of glycogen synthase kinase 3 beta via phosphorylation of Ser-389. A decrease in adult neurogenesis may be connected to depression since stressful conditions decrease hippocampal neurogenesis, whereas antidepressant treatment enhances neurogenesis [32]. Intracerebroventricular transfer of adiponectin advances peripheral insulin sensitivity and glucose homeostasis [33], suggesting that central actions of adiponectin may also affect metabolic diseases.

### 4.2. Adiponectin Functions in the Liver

Adiponectin contributes to the control of glucose uptake and lipids metabolism, by reducing gluconeogenesis and enhancing glycolysis and fatty acid oxidation in the liver. These metabolic effects happen through interactions with AdipoR1 and hepatic AdipoR2. These two receptors trigger two different signaling pathways; AdipoR1 activates AMPK and AdipoR2 enhances the PPAR-α cascade [34]. Activated AMPK inhibits phosphoenolpyruvate carboxykinase (PEPCK) and glucose 6-phosphatase (G6Pase) transcription, which leads to gluconeogenesis reduction [35]. In addition, AMPK inhibits lipid synthesis through acetyl-CoA carboxylase inhibition (ACC), which catalyzes fatty acid precursor biosynthesis (malonyl-CoA). Malonyl-CoA is also a potent inhibitor of carnitine palmitoyl transferase I (CPT-I), the enzyme that controls long chain fatty acid transfer into the mitochondria. Therefore, adiponectin promotes lipid oxidation and inhibits triglyceride synthesis in the liver through AMPK signaling [36,37]. Activated AMPK phosphorylates Ser372 of the sterol regulatory element binding protein 1c (SREBP-1c), and leads to the suppression of SREBP-1c, a master regulator of fatty acid biosynthesis. In the liver, free fatty acid oxidation is also due to the signaling cascade triggered by PPAR-α, which cooperates with AMPK in enhancing fatty acid oxidation [34].

As can be seen from the mentioned data, insulin and adiponectin have various and different metabolic effects in the liver, which are listed in Table 1. In summary, insulin stimulates lipogenesis and glycolysis and glycogen synthesis, whereas adiponectin stimulates fat oxidation.

### 4.3. Adiponectin Functions in the Muscle

Plasma concentrations of adiponectin inversely correlate to weight, central obesity, risk of types 2 diabetes (T2D), and insulin resistance in humans [38]. Adiponectin promotes insulin sensitivity [39], fatty acid oxidation through activation of AMPK, p38-MAPK, and PPAR-α, and also enhances glucose uptake in the skeletal muscle [40]. AMPK is a serine/threonine protein kinase that controls glucose concentration and lipid metabolism as a primary cell energy balance sensor in mammalian cells. Adiponectin activates AMPK through interaction between AdipoR1 and APPL1-compatible protein. AMPK activation induces fatty acid oxidation and glucose entry to the muscle cells. It has been shown that adiponectin enhances APPL1-dependent protein LKB1 transfer from the nucleus to the cytosol, which leads to the activation of AMPK [35]. Adiponectin also triggers CaMKK by stimulating intracellular release of Ca^2+^ through a PLC-dependent mechanism, which activates AMPK. AMPK activation result in enhanced glucose entry to the muscles and fatty acid oxidation [35]. An increase in glucose uptake and fatty acid oxidation can also happen through enhancing PPAR in the response to the increased levels of adiponectin [41].

### 4.4. Adiponectin Functions in the Heart

Adiponectin has beneficial effects on the heart through APPL1-AMPK cascade. It protects the heart through various mechanisms. Adiponectin has been shown to increase translocation of cluster of differentiation 36 (CD36) and absorption of fatty acids along with increased insulin-stimulated glucose uptake and phosphorylation of Akt, in cardiomyocytes. Adiponectin also increases AdipoR1 interactions with APPL1, then APPL1 binds to AMPK-α2 result in acetyl-CoA carboxylase (ACC) phosphorylation and its inhibition, which leads to increased oxidative phosphorylation in heart tissue [42].

### 4.5. Adiponectin Functions in the Kidney

**Figure 3:**
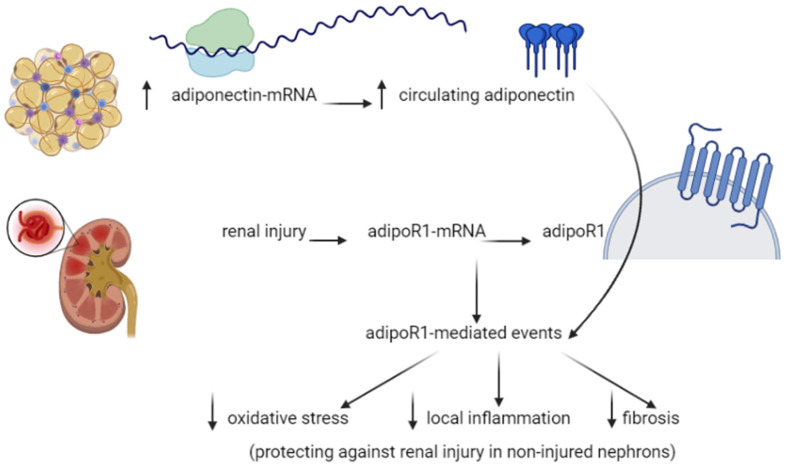
The proposed role of adiponectin in kidney pathophysiology [44].

Both AdipoR1 and R2 are expressed in the kidneys [43]. Adiponectin can protect kidneys from albuminuria in mice models [44]. It has also been shown that adiponectin has antioxidant effects on the kidneys through the activation of AMPK related pathways, so it can reduce inflammation at the kidney level (Figure 3) [44]. Tsioufis et al. (2005) estimated the levels of adiponectin in non-diabetic hypertensive men in relation to microalbuminuria. They found that microalbuminuria was associated with lower adiponectin levels [45].

### 4.6. Adiponectin Function in the Bone

Increases in body fat are usually accompanied with an increase in bone mass, and low body fat is accompanied with low bone mineral density and fractures. Interactions between bone and adipose tissue are not only due to the mechanical load of adipose tissue, but also through the release of the cytokines from adipose tissue and its effects on the bone [46]. Adiponectin has been considered as one of the mediators of the fat-bone relationship. There is a negative relation between blood adiponectin concentrations and bone mineral density. AdipoR1 and R2 are synthesized in human primary osteoblasts and in bone marrow macrophages, and they stimulate osteoclasts differentiation [46]. Adiponectin functions in different body organs are summarized in Table 2.

## 5. Adiponectin and Diseases

### 5.1. Adiponectin and Diabetes

In general, inactive lifestyles lead to obesity [47]. According to the World Health Organization (WHO), more than 1.4 billion persons are overweight and over 500 million people are obese. Obesity is considered as a risk factor for diabetes, and ultimately inability and increased mortality in the elderly [48]. Diabetes mellitus is characterized by high glucose levels in the blood and is one of the serious conditions that affect other organs, such as kidneys, eyes, blood vessels, and nerves. Patients with diabetes mellitus type 2 represent about 90% of all diabetic patients [38].

Obesity induces insulin resistance, and consequently, type 2 diabetes, which is associated with fat accumulation and malfunction of insulin [49]. Insulin resistance is defined as insulin inability to perform metabolic and vascular tasks in target tissues [48]. The percentage of people with type 2 diabetes is rising worldwide, approximately 6.5% (285 million subjects) in 2010, which is expected to rise to 7% in 2030 (439 million) [49]. Understanding the cause of such an increase is the most important step toward effective control and management. While a lot of cases are due to high food intake, unhealthy diet and inactive lifestyle, there is also an emerging body of evidence pointing at genetic factor influences [49].

Adipose tissue secretes proteins, such as adiponectin, to control insulin sensitivity, increase fat metabolism, regulate glucose tolerance, and modify homeostasis to protect individuals from diabetes [50]. Therefore, adiponectin is considered as one of the strongest markers of type 2 diabetes mellitus [20,51]. The effect of adiponectin on insulin sensitivity was first reported in mice [52]. Plasma adiponectin levels have a negative correlation with insulin resistance development and type 2 diabetes mellitus [53]. The primary source of evidence for the capability of adiponectin to enhance glucose tolerance was that in a model of diabetic rats, taking a single dose of adiponectin resulted in significant reductions in blood glucose levels [54]. There is an association between ADIPOQ polymorphisms and type 2 diabetes mellitus [49]. Adiponectin affects insulin sensitivity in diabetic patients through the following direct and indirect mechanisms:

1. Adiponectin decreases the amount of adipose tissue triglyceride and controls insulin signaling; moreover, adiponectin enhances expression of fatty acids transmitter molecules such as CD36 and also acyl-coenzyme oxidase, and therefore, plays an important role in skeletal muscle triglyceride [40]. It has been reported that the triglyceride content is increased with the activation of insulin-stimulated phosphatidylinositol [PI] 3-kinase, followed by the replacement of glucose-4 transporter (GLUT-4) and increased glucose uptake, leading to insulin resistance; therefore, decreased triglyceride content in the muscle will probably help improve the transmission of the insulin signaling pathway;

2. Adiponectin activates PPAR-α receptor phosphorylation activator: adiponectin enhances fatty acids oxidation and energy consumption by activating PPAR-α, which leads to a reduction in the triglyceride content in liver and muscle, which ultimately increases insulin sensitivity [55];

3. Adiponectin activates AMPK cascade. In summary, adiponectin stimulates AMPK phosphorylation and activation in skeletal muscle, which stimulates beta-oxidation [55].

### 5.2. Adiponectin and Cancer

Adiponectin concentration varies in different conditions, but its levels are decreased in several types of cancer. Adiponectin anticancer signaling pathways are various and complex [55]. Adiponectin may activate or inhibit these pathways when it is presented directly or indirectly to AdipoR1/2. Adiponectin activates AMPK, Fas ligand and JNK, whereas it inhibits Wnt, STAT3, PI3K/Akt, USP-2, and ERK1/2. Adiponectin also promotes ceramidase activity, increasing the conversion of ceramide to S1P [56].

Adiponectin is considered as an important link between colon cancer and obesity [57,58]. It was also observed that low expression of adiponectin and high expression of its receptors may be linked with invasive breast cancer [59,60]. It has also been shown that decreased levels of Adiponectin are accompanied with an increased risk of uterine cancer, especially in women below the age of 65, regardless of BMI, ethnicity, IGF, and other known factors [61]. Adiponectin inhibits the development of gastric cancer, suppresses liver tumors, and decreases the risk of liver cancer [62,63].

### 5.3. Adiponectin and Cardiovascular Diseases

Serum adiponectin concentrations are negatively associated with obesity, diabetes (type 2), and cardiovascular disease [64]. Negative correlation between adiponectin and cardiovascular disease was seen in several studies [65,66]. Hypo-adiponectinemia also increases the risk of high blood pressure and cardiomyopathy in diabetic patients [67,68,69]. Adiponectin helps reduce vascular dysfunction through enhanced release of NO and decreased expression of sticky particles [70]. On the other hand, blood levels of adiponectin are significantly increased in heart failure. Therefore, it is still controversial to consider adiponectin as a marker of cardiovascular disease [71].

A recently discovered adipokine, nephroblastoma overexpressed CCN3 (NOV/CCN3), is a multifunctional protein of the CCN family which is involved in many pathophysiological processes, including inflammation, interstitial fibrosis, and renal tissue damage and repair [72]. NOV also modulates cell proliferation, cell adhesion, and the subsequent induction of pro-inflammatory cytokines and chemokines in human cardio-metabolic patients. Elevated NOV is attributed to increased obesity, plasma triglycerides, and C-reactive protein [73]. Impairment of mitochondrial energetics increases the levels of reactive oxygen species (ROS) being produced and the resultant oxidative stress is considered a primary risk factor in the development of diabetic cardiomyopathy [74]. Obesity and ROS induction contribute to an increase in NOV and reduction in heme oxygenase-1 (HO-1) levels [75,76]. Singh et al. indicated that obesity and oxidative stress are accompanied with increased NOV levels and inflammation associated with increased release of TNF- α and IL-6 and a decrease in HO-1 and peroxisome proliferator-activated receptor gamma coactivator-1 alpha (PGC-1α) [77]. PGC-1α is a master regulator of mitochondrial biogenesis, which regulates respiratory chain complexes and ATP synthases and its levels in cardiomyocytes are critical to mitochondrial polarization, adequate output of ATP for cardiac energy supply [78]. In summary, obesity-induced oxidative stress down-regulates PGC-1α and HO-1 and increases mitochondrial dysfunction and insulin resistance, which finally leads to cardiomyopathy [78].

### 5.4. Adiponectin and Alzheimer’s Disease

Alzheimer’s disease is a disorder of the central nervous system affecting the elderly over 65 years of age [79]. More than 5 million people live with Alzheimer’s disease, and mortality rates have increased by 89% in the United States since 2000 [80]. Research has shown correlation between Alzheimer’s disease and insulin resistance in the brain [81,82,83]. Moreover, increased body weight is inversely related to adiponectin levels [83]. Therefore, it has been shown that losing weight in patients with Alzheimer’s disease can influence serum adiponectin [84]. Alzheimer’s disease drugs, such as acetylcholinesterase inhibitors, may increase adiponectin levels [85]. Thiazolidinediones, which are PPAR-γ agonists, and lipid-lowering drugs, such as niacin and some statins, can also increase adiponectin levels [86,87,88,89,90]. In 2017, Ng and Chan stated that reducing the levels of adiponectin or reducing the adiponectin signaling activity promote the progression of Alzheimer’s disease and cause cognitive impairment. This association is closely connected to impaired insulin signaling pathway and decreased brain insulin sensitivity [91]. Compared with high expression of adiponectin in plasma, the levels of adiponectin are very low in nerve cells [92]. A clinical study indicated that decreasing plasma levels of adiponectin is a risk factor for women with Alzheimer’s disease [93]. Letra et al. suggested that increase in adiponectin levels might have a neuroprotective effect on Alzheimer’s disease [94]. 

### 5.5. Diet and Supplements

Healthy diet enhances the levels of adiponectin [95]. The Mediterranean diet, as a popular and healthy diet, has high levels of whole grains, low-glycemic carbohydrates, fibers, unsaturated fats, vegetables, and fruits, a balanced consumption of dairy products and fish, and lower ingestion of red meat, confectionery, and saturated fatty acids. An increasing body of research showed increased adiponectin levels in consumers of Mediterranean diet [96]. Patres et al. (2016) concluded that 122 diabetic patients (type 1) with high intake of saturated fatty acid had decreased adiponectin levels [53], but a diet with high intake of unsaturated fats was able to increase their adiponectin levels [97,98]. Unsaturated fatty acids stimulate the release of adiponectin through gamma peroxidation activation. Eicosapentaenoic and docosahexaenoic acids activate the PPAR-γ pathway, which stimulates the expression of adiponectin mRNA in adipocytes [99]. Consuming low glycemic index foods, such as fruits and vegetables, plays a key role in protecting the body against inflammation caused by metabolic syndromes and cardiovascular diseases by increasing adiponectin levels [100]. Weight loss diets [101] and daily use of yellowish herbs [102] also increase adiponectin. Barbosa et al. (2017) found that consuming 3 grams fish oil for 2 months led to 23% increase in adiponectin levels in patients with cardiovascular diseases [103]. Two meta-analysis and review papers suggested that taking omega-3 as a supplement and eating omega-3 rich foods increases adiponectin and subsequently improves metabolic factors in the patients with cardiovascular disease and type 2 diabetes, although optimal dosage is still controversial [103,104,105,106]. Silva et al. (2011) showed a 60% increase in levels of adiponectin with a daily intake of fish and other sources of omega-3 fatty acids [107]. Research showed that the consumption of omega-3 in people with the lowest basal levels of adiponectin had the greatest effect on lipid profiles, whereas in individuals with moderate basal adiponectin, the major effect was on oxidative profiles. In people with high levels of adiponectin, omega-3 uptake had the highest effect on glucose metabolism [103].

The mechanisms through which omega-3 fatty acids increase adiponectin levels are as follows:

(1) Omega-3 consumption causes fat loss and, since adiponectin has a negative correlation with body fat, this increases adiponectin [103];

(2) The levels of TNF-α and interleukin-6 (IL-6) are decreased by the inhibitory activity of IκB-α. This binding inhibitor protein is associated with the Nuclear Factor Kappa-B molecule (NF-κB). When IκB-α begins to phosphorylate, it simultaneously undergoes a degradation of the protein bound to the ubiquitin system and eventually deactivates NF-KB. This results in NF-KB-dependent gene transcription, such as TNF-α and IL-6. These cytokines decrease the expression of the ADIPOQ gene and ultimately inhibit the synthesis and secretion of adiponectin, but omega-3 supplements can counteract/revert such molecular events, by preventing IκB-α phosphorylation. Following this, adiponectin is synthesized and released [108];

(3) Via PPAR-γ cascade. Activation of omega-3 fatty acids are another mechanism naturally associated to PPAR-γ receptor pathway [103].

Relations between adiponectin and human diseases are summarized in Table 3.

## 6. Conclusions

Adiponectin is a cytokine that is secreted from adipose tissues (adipokine). Available evidence suggests that adiponectin is involved in a variety of biological processes and biochemical events, such as lipid metabolism, energy regulation, inflammation, and insulin sensitivity. It has a protective effect on neurons and neural stem cells. Adiponectin levels have been reported to be negatively correlated with cancer, cardiovascular disease, and diabetes, and have been shown to be affected by nutrition. The present review has comprehensively overviewed the role of adiponectin in a range of diseases, suggesting that adiponectin can be a useful biomarker of these disorders and can be the target for monitoring the effectiveness of different preventive and treatment interventions.

## Figures and Tables

**Table 1:** Insulin and Adiponectin effects on the liver.

	Insulin	Adiponectin
Lipogenesis	↑	↓
Fat oxidation	↓	↑
Glycogenolysis	↓	↓
Gluconeogenesis	↓	↓
Glycolysis	↑	-
Glycogen synthesis	↑	-

**Table 2:** Adiponectin functions in different body organs.

Organ	Adiponectin Functions
Brain	❖ Insulin-sensitizing, anti-inflammatory, angiogenic, and vasodilatory properties ❖ Can cross the brain barrier and be detected in the cerebrospinal fluid ❖ Controls important brain functions such as energy homeostasis, hippocampal neurogenesis, and synaptic plasticity ❖ Controls neurogenesis and synaptic plasticity ❖ AdipoR1 is expressed primarily in the hippocampus ❖ May have a significant effect on cognitive functions
Liver	❖ Controls glucose and lipids metabolism; reduces gluconeogenesis and enhances glycolysis and fatty acid oxidation ❖ Reduces fat accumulation through activation of SIRT1-AMPK pathway ❖ Inhibits gluconeogenesis, reduces the levels of insulin in the bloodstream, which decreases lipogenesis and increases fatty acids oxidation
Muscle	❖ Promotes insulin sensitivity and fatty acid oxidation and enhances glucose uptake in skeletal muscles ❖ Enhances APPL1-dependent protein LKB1 transfer from nucleus to the cytosol, which leads to the activation of AMPK
Heart	❖ Protects heart through following mechanisms: Increases CD36 expression and fatty acid absorption, as well as insulin-stimulated glucose transport and Akt phosphorylation, in cardiomyocytes Increases interactions between AdipoR1 with APPL1 and APPL1 binding to AMPK-α2 results in ACC phosphorylation and inhibition which subsequently increase in fatty acids oxidation
Kidney	❖ Beneficial effects on the kidneys and also protects from albuminuria in mice models ❖ Anti-inflammatory and antioxidant effects through the activation of protein-kinase-activated AMPK pathway
Bone	❖ Mediator of fat-bone relationship ❖ Negative relation between blood adiponectin concentrations and bone mineral density ❖ AdipoR1 and R2 are expressed in human primary osteoblasts and in bone marrow macrophages and stimulate osteoclasts differentiation

**Table 3:** Adiponectin and human diseases.

Disease	Effect on Adiponectin
**Diabetes**	❖ Controls insulin sensitivity, increases fat metabolism, regulates glucose tolerance, and modifies homeostasis to protect individuals from diabetes ❖ Plasma adiponectin levels have a negative correlation with type 2 diabetes mellitus ❖ Adiponectin affects insulin sensitivity through the following mechanism: Decreases the amount of adipose tissue triglycerides and regulates insulin signaling Activates PPAR-α receptor phosphorylation activator Activates AMPK cascade ❖ Enhances fatty acids oxidation and energy consumption by activating PPAR-α
**Cancer**	❖ Its levels are decreased in several types of cancer ❖ Low expression of adiponectin and high expression of its receptors may be associated with invasive breast cancer ❖ Low levels of adiponectin are associated with an increased risk of uterine cancer, especially in women below the age of 65 years
**Cardiovascular Diseases**	❖ High plasma levels of adiponectin are associated with reduction in the risk of coronary artery disease ❖ Hypo-adiponectinemia also increases the risk factor of high blood pressure and cardiomyopathy in diabetic patients ❖ Adiponectin levels are significantly increased in heart failure ❖ Reduces vascular dysfunction by increasing NO production and reducing the expression of sticky molecules
**Alzheimer’s disease**	❖ Reduction in the levels of adiponectin or reduction in the adiponectin signaling activity can promote the progression of Alzheimer’s disease and cause cognitive impairment ❖ Decreased plasma levels of adiponectin are a risk factor for women with Alzheimer’s disease ❖ May have a neuroprotective effect on Alzheimer’s disease
